# Buffalo Academy of Medicine

**Published:** 1902-12

**Authors:** N. L. Burnham


### Buffalo Academy of Medicine.

Reported by N. L. BURNHAM, M. D., Secretary.

*Section on Medicine, October 14, 1902.*

Dr. George Dock, Ann Arbor, Mich., read a most interesting paper on Jenner's works and their value in the modern study of smallpox.

The speaker gave an account of Edward Jenner, his character, methods of work and steps he followed in the discovery of the efficacy of vaccination. This was based on a study of the first editions of Jenner's various pamphlets, a collection of these having been loaned to the speaker by Dr. Wm. Osler. Lantern slides of title pages, illustrations and striking paragraphs from Jenner's works were exhibited. The speaker then described the spread of vaccination in various parts of the world; the decrease and subsequent recrudescence of smallpox; the discovery of the need of revaccination, culminating in the events of 1870 and 1871 in France and Germany, and the epoch-making vaccination laws passed by the German empire. A plea was made for general vaccination and revaccination on the ground that the operation was of more importance to the community than to the individual; also for the control of the production and distribution of vaccine material and for the systematic recording of the results of vaccination. Some of the difficulties in the way of these proposals were shown to be apparent rather than real. In the meantime the necessity of careful work on the part of physicians was emphasised.

DISCUSSION.

Dr. DeLancey Rochester in discussing the paper pointed out the evils of commercialism in the production of a reliable virus. He favors state control of the manufacture of virus.

Dr. H. R. Hopkins advocated state control of vaccination and a national organisation of sanitation—a secretary of health. He drew attention to the recent failure of cleanliness in Cleveland as instituted by the mayor to prevent the spread of smallpox.

Dr. Irving P. Lyon remarked that New York city manufactures its own virus.

Dr. Charles G. Stockton complimented the speaker upon an honest statement of the facts relative to the works of Jenner. Physicians should be outspoken and laymen instructed as to the necessity of vaccination in order to overcome the harm done by antivaccinationists.

Dr. Roswell Park stated that it was of interest to note that previous to vaccination, inoculation was introduced from the Orient by Lady Worthy Montague into England. He also referred to the correspondence between John Hunter and his pupil Jenner. The latter performed many arduous duties gratuitously, and added many specimens to the Hunterian collection.

Dr. Arthur W. Hurd referred to the assistance rendered the cause of vaccination by Thomas Jefferson, then President of the United States, by having his entire household vaccinated by a Boston physician, who introduced into America this method of preventing smallpox.

Dr. DOCK closed the discussion, expressing his thanks for the reception and hearty discussions of his paper by the Academy members. He stated that previous to vaccination inoculation was prohibited in the United States. The state control of vaccination is desirable.

Meeting adjourned at 10.30 p.m. Fellows present, 44.

---

*Stated Meeting of the Academy, November 3, 1902.*

REPORTED BY F. W. McGUIRE, M. D., Secretary.

Meeting called to order by the president, Dr. HERMAN E. HAYD at 8.50 p.m.

The minutes of the last stated meeting, held September 9, 1902, were read and approved.

The council recommended the election of Drs. Adolph H. Urban, 523 Franklin Street, and Theodore V. Bauer, 437 Genesee Street. They were duly balloted for and elected members of the Academy.

The program of the evening was provided by the surgical section. It consisted of a paper read by Dr. ROSWELL PARK, entitled, The physics and therapeutic value of the (Röntgen) cathode and ultra-violet rays, with certain references to the electro-magnetic theory of light, with exhibition of new instruments for phototherapy.

The paper was discussed by Drs. Blaauw, Starr, Dunham, Gaylord, Mann, Rochester, Clewes, Bayliss and Hayd.

Adjourned at 10.30 p.m. Attendance, 108.

---

*Section on Medicine, November 11, 1902.*

REPORTED BY N. L. BURNHAM, M. D., Secretary.

The regular meeting of the Medical Section of the Academy of Medicine was held November 11, 1902, in the Academy rooms, Buffalo Public Library Building.

Meeting was called to order at 8.45 p.m., the chairman, Dr. ALBERT E. WOEHNERT, presiding.

The minutes of the previous meeting were read and approved.

Dr. George M. Gould, editor of *American Medicine*, Philadelphia, read a paper entitled,

MEDICAL DISCOVERIES BY NON-MEDICAL MEN.

The paper of Dr. Gould was to show how frequently medical discoveries, instruments, and the like, have been led up to by a graded series of preliminary steps, and that non-medical men have often been the discoverers. Even animals have considerable medical and surgical art, and savages and primitive men have displayed remarkable ability in this way, and have made many important discoveries, which were described. The laryngoscope and vaccination were discovered by non-medical men. Moses was the greatest sanitarian that ever lived, and much could be learned of him in hygiene today. These facts are encouraging rather than the reverse, as even the simplest country practitioner, seeing individual diseases and each patient's case differing from all others, has always before him the possibility of individual discovery.

DISCUSSION.

Dr. A. L. Benedict, in opening the discussion, drew attention to the sites of the Indian villages in the vicinity of Buffalo. They are near water, located on high ground, while their burying grounds are far removed from their camps, in order to prevent pollution of water supply.

Dr. W. Van Peyma pointed out that long ago the use of the scorched cloth upon the navel of the new-born baby was the practice of antisepsis without the knowledge of why it was done.

The paper was further discussed by Drs. Blaauw and Clewes, the latter referring to Pasteur, Liebig, and Fischer as non-medical men.

The second paper of the evening was read by Dr. William P. Spratling, superintendent of Craig Epileptic Colony, Sonyea, N. Y., on

HOW EPILEPTICS ARE CARED FOR AND TREATED AT SONYEA.

Dr. Spratling referred to the fact that the best way to convey an idea of how epileptics are cared for and treated at Sonyea, was by showing a series of sixty-eight lantern slides, but before doing this he would speak briefly on what the colony system meant.

He referred to two kinds of colonies: one for selected cases only, the other for all epileptics, save those continuously and actively insane. There is no colony of the former type in this country just now, the only one being the colony at Chalfont St. Peter, near London. The Craig Colony is an illustration of one of the mixed type, since it receives all epileptics save those insane.

Before building or caring for the epileptic in an institution of any kind, his peculiarities must be studied, especially with a view to educate him along proper lines and to provide for his proper classification. Epilepsy being essentially a disease of the brain, in all cases where the disease is genuine, the best form of education is that which recognises manual training to the greatest extent. At the same time some intellectual education is necessary.

Epileptics do all kinds of work at Sonyea, from needle-work and house painting to engine driving. The forms of treatment are varied and are mapped out under these heads: medical, surgical, dietetic and moral. Nothing is lost sight of in the treatment of the case that may be of benefit.

The following summary was given of the treatment of epileptics at Sonyea:

1. It effects cures in a larger proportion of cases than can be effected under any other form of treatment, notwithstanding the fact that few cases are sent to the colony before the disease is essentially chronic.

2. It brings about a reduction in the frequency and severity of attacks in the majority of all cases, a large per cent. being sufficiently improved to permit them to go into the outside world to earn a living.

3. It provides special education for a class in the special manner they require it to make them self-helpful, this being something they cannot get outside of the colony.

4. It promotes individual happiness in a large proportion of cases, due to the patient's living in an atmosphere of congeniality, an atmosphere saturated with a fellow-feeling and desire to help each other.

5. It provides skilled forms of treatment by those who do no work but this, and the opportunity for scientific research that can nowhere else be found and that should be here done for the benefit of all who suffer in this terrible way.

6. Segregating epileptics in this way has a decided economic value, for as long as they are kept in proper seclusion, that seclusion being at the same time most beneficial to the epileptic, it shuts off absolutely the probability of that epileptic handing down a defective or an epileptic progeny, something that all epileptics are much too prone to do. The presence of an epileptic in the marriageable world is like a bank account at compound interest—it keeps increasing its kind.

DISCUSSION

Dr. William C. Krauss opened the discussion, pointing out the necessity of sending patients to Sonyea during the acute stages of epilepsy, if we wish to obtain the best results. He moved a vote of thanks be tendered the readers of the evening's papers. Motion was seconded by Dr. Charles G. Stockton. Carried.

Dr. James W. Putnam has visited Craig Colony. The epileptics at Sonyea are not a despondent class. Occupation is necessary to successful treatment.

Dr. Allen Jones complimented the speaker upon his valuable and most interesting paper. He requested enlightenment upon the belladonna treatment if used at Sonyea. Also whether in all cases active treatment is carried out, or do some cases go on from month to month without treatment.

Dr. Spratling, in closing the discussion, said that anything and everything that offers relief is used at Sonyea. Drs. Gould and Bennett had refracted many cases with hopes of relief from the epileptic seizures. The belladonna treatment has been used. It is beneficial in some cases; useless in others. Bromides suppress the attacks, but do not cure. Salt starvation is also practised. Bromopin is a new remedy being used at present, which gives very favorable results. It is very expensive, and is best given in an emulsion. Ten grains of this drug are as strong as forty or fifty grains of bromide.

Upon request Dr. Gould, of Philadelphia, referred to his work at Sonyea on eye refraction. He stated it was too early to make any statement as to results. Certainly the correction of eye-strain must relieve many of the accompanying symptoms, as headache, and the like. Dr. Gould was led to try refraction upon Sonyea epileptics on account of the marked improvement resulting in one of his private cases.

Meeting adjourned at 11 p.m. Fellows present, 65.

